# Toll-like receptors 2 and 4 differentially regulate the self-renewal and differentiation of spinal cord neural precursor cells

**DOI:** 10.1186/s13287-022-02798-z

**Published:** 2022-03-21

**Authors:** Marina Sanchez-Petidier, Consuelo Guerri, Victoria Moreno-Manzano

**Affiliations:** 1 Neuronal and Tissue Regeneration Laboratory, Prince Felipe Research Institute, Valencia, Spain; 2 Neuropathology Laboratory, Prince Felipe Research Institute, Valencia, Spain

**Keywords:** Neural precursor cells, Self-renewal, Cell differentiation, Spinal cord, Toll-like receptors, Neurogenesis

## Abstract

**Background:**

Toll-like receptors (TLRs) represent critical effectors in the host defense response against various pathogens; however, their known function during development has also highlighted a potential role in cell fate determination and neural differentiation. While glial cells and neural precursor cells (NPCs) of the spinal cord express both TLR2 and TLR4, their influence on self-renewal and cell differentiation remains incompletely described.

**Methods:**

TLR2, TLR4 knock-out and the wild type mice were employed for spinal cord tissue analysis and NPCs isolation at early post-natal stage. Sox2, FoxJ1 and Ki67 expression among others served to identify the undifferentiated and proliferative NPCs; GFAP, Olig2 and β-III-tubulin markers served to identify astrocytes, oligodendrocytes and neurons respectively after NPC spontaneous differentiation. Multiple comparisons were analyzed using one-way ANOVA, with appropriate corrections such as Tukey's post hoc tests used for comparisons.

**Results:**

We discovered that the deletion of TLR2 or TLR4 significantly reduced the number of Sox2-expressing NPCs in the neonatal mouse spinal cord. While TLR2-knockout NPCs displayed enhanced self-renewal, increased proliferation and apoptosis, and delayed neural differentiation, the absence of TLR4 promoted the neural differentiation of NPCs without affecting proliferation, producing long projecting neurons. TLR4 knock-out NPCs showed significantly higher expression of Neurogenin1, that would be involved in the activation of this neurogenic program by a ligand and microenvironment-independent mechanism. Interestingly, the absence of both TLR2 and TLR4, which induces also a significant reduction in the expression of TLR1, in NPCs impeded oligodendrocyte precursor cell maturation to a similar degree.

**Conclusions:**

Our data suggest that Toll-like receptors are needed to maintain Sox2 positive neural progenitors in the spinal cord, however possess distinct regulatory roles in mouse neonatal spinal cord NPCs—while TLR2 and TLR4 play a similar role in oligodendrocytic differentiation, they differentially influence neural differentiation.

**Supplementary Information:**

The online version contains supplementary material available at 10.1186/s13287-022-02798-z.

## Background

The Toll-like receptor (TLR) family in mammals includes thirteen members—mice and humans express TLR1-9, while mice additionally express TLR11-13, and humans additionally express TLR10 [1]. TLRs respond to a large number of ligands, including pathogen-associated patterns (PAMPs) such as lipopolysaccharide (LPS), nucleic acids, and proteins such as extracellular matrix components (fibrinogen, fibronectin, and hyaluronic acid fragments) or endogenous damage-associated patterns (DAMPs), which are released following injury [2, 3]. Interestingly, *Drosophila* TLRs also recognize neurotrophins such as brain-derived neurotrophic factor (BDNF) and nerve growth factor (NGF) [4]. Following ligand binding, TLRs activate their corresponding signaling components by Myeloid differentiation primary response 88 (MYD88)-dependent and TIR-domain-containing adapter-inducing interferon-β (TRIF)-dependent pathways. All TLRs recruit MyD88 except for TLR3, which utilizes TRIF to mediate signaling. These signaling pathways have been described for the classical role of TLRs linked to innate immunity that help to orchestrate the immediate and specific adaptive immune response by cytokine and chemokine delivery activating antigen-presenting cells such as macrophages, microglia, or dendritic cells (reviewed in Kawai and Akira [5]).

Interestingly, studies have demonstrated that mammalian TLRs and their Drosophila homologs trigger cell-autonomous processes independent of ligand activation during embryogenesis [3] and adult neurogenesis [6–8]. Constitutive expression of several TLR family members occurs in astrocytes [9], oligodendrocytes [10], neurons [11, 12], and neural precursor cells (NPCs) [13]. Additionally, recent research has suggested a role for TLR2 and TLR4 in supporting neuronal morphogenesis and plasticity under physiological [3, 7] and pathological conditions [14–17]. TLR2 forms heterodimers with TLR6 and TLR1 to trigger MyD88-dependent signaling, while TLR4 employs both MyD88-dependent and TRIF-dependent signaling pathways [5, 18]. Rolls et al. studied the function of TLR2 and TLR4 in mouse adult hippocampal NPCs, finding the requirement of TLR4 and MyD88 for astrocytic differentiation and TLR2 in neural differentiation in healthy individuals [7]. TLR4 activation following exposure to LPS, an inflammatory mediator, negatively impacts hippocampal NPC proliferation, suggesting a detrimental effect of TLR4 on neurogenesis [7]; however, TLR4 activation in a model of ischemic stroke-induced NPC proliferation and survival within the subventricular zone promoted neural differentiation of migrating neuroblasts, suggesting a role in endogenous brain repair [19]. Furthermore, Graselli et al. reported a requirement for TLR4 expression in the self-renewal and neuronal/oligodendrocytic differentiation of NPCs from the human fetal brain [20]. Thus, current evidence suggests contrasting roles for TLRs in NPCs, which could derive from ligand-specific activation pathways and/or to cell- and species-specificity.

While we have evidence for the role of TLRs in brain development and morphogenesis, we know little regarding their role in spinal cord NPCs. In the healthy adult human and mouse spinal cord, studies have highlighted *TLR2* and *TLR4* as the most highly expressed of the TLR family [21, 22], and the significant overexpression of *TLR2* and *TLR4* following spinal cord injury (SCI) in experimental rodent models [23, 24]. The activation of TLR2 signaling by intrathecal or intramedullary injection of zymosan in the spinal cord produces demyelination, axonal damage, and astrocytic activation [25], which has been attributed to the activation and infiltration of resident microglia and the activation of circulating monocytes, mimicking the inflammatory responses observed after SCI [25, 26]. However, studies have noted that the absence of TLR2 [24] or TLR4 [27] limits spontaneous regeneration, impairs remyelination, and sustains locomotor deficits after SCI due, at least in part, to reduced iron metabolism and unbalanced growth factors delivery, which impair oligodendrocyte precursor cell (OPC) maturation [27].

In our new study, we describe the role of TLR2 and TLR4 in the proliferation and differentiation of neonatal spinal cord NPCs. While our results suggest the requirement for both TLR2 and TLR4 to maintain Sox2 positive precursors, they possess distinct roles in neuronal differentiation. While a lack of TLR2 reduce neuronal differentiation and promotes NPC self-renewal, TLR4 loss prompts the increased differentiation of Neurogenin1-expressing neurons.

## Methods

### Study design

Wild type (WT), TLR2^−/−^ and TLR4^−/−^ C57BL/6J mice (kindly provided by Dr. S. Akira, Osaka University, Suita, Japan [28]) were used to comparatively evaluate the influence of both TLRs in NPC self-renewal and cell differentiation. First, spinal cords from at least eight different P4 neonatal mice of each strain, were evaluated by histological analysis. Then, for in vitro experimentation, at least three independent experiments were performed by using a pool of spinal cords of neonates from the same litter, to generate every primary neurosphere-like cultures. NPC identity was evaluated under growth conditions, in the presence of mitogen factors for one day, or in the absence of mitotic factors after one week on culture, in adherent conditions. The percentage of each cell population, astroglial, oligodendrocytes and neurons was evaluated by specific immune staining.

### Spinal cord tissue isolation

Spinal cords from WT, TLR2^−/−^, or TLR4^−/−^ C57BL/6J mice at the P4 postnatal stage were dissected from cervical to lumbar segments for histological studies or NPC primary cell culture. Spinal cords used for histological analysis were immediately fixed in 4% paraformaldehyde (PFA) for 4 h after dissection.

### NPC culture and treatment

Following the removal of the overlying meninges and blood vessels, dissected spinal cords were placed in fresh washing medium (WM: DMEM/F12 supplemented with 100 units/ml penicillin, 100 µg/ml streptomycin, 5 mM HEPES buffer, 0.125% NaHCO3, and 0.09% glucose), cut into 1 mm^3^ pieces, and disaggregated to single-cells in growth medium (GM: NeuroCult™ Proliferation Medium supplemented with NeuroCult™ Proliferation Supplement [STEMCELL Technologies, USA] including 20 ng/ml epidermal growth factor [EGF, Invitrogen], 20 ng/ml basic fibroblast growth factor [bFGF, Invitrogen], 1X penicillin/streptomycin, and 2 µg/ml heparin [Sigma]) by passing through a 200 µl micropipette tip twenty times. NPCs were selected based on their capacity to form neurospheres under non-adherent conditions and then cultured in ultra-low attachment plates in GM at 37 °C and 5% CO_2_ in a saturated humid atmosphere.

For the quantification of neurosphere number and size, 5 × 10^3^ NPCs were seeded in 96 ultra-low attached/well plates for 48 h, and then phase-contrast images analyzed using Image J. For proliferation analysis and downstream signaling activation, neurospheres were disaggregated with StemPro Accutase Cell Dissociation Reagent (ThermoFisher) for 5 min at 37 °C and seeded onto Matrigel™ coated coverslips (diluted 1/20 times in DMEM/F12). NPCs cultured at 4 × 10^4^ cells/cm^2^ as a monolayer in GM without FGF and EGF were treated with LPS (50 ng/ml, Sigma-Aldrich) for 30 or 60 min.

For spontaneous differentiation analysis, NPCs at 4 × 10^4^ cells/cm^2^ were seeded on Matrigel™ coated coverslips and cultured for seven days. Cells were cultured in GM without EGF and FGF for the first two days, after which GM replaced by Differentiation Medium (DM: DMEM/F12 supplemented with 100 units/mL penicillin, 100 µg/ml streptomycin, 2 mM l-glutamine, 5 mM HEPES buffer, 0.125% NaHCO3, 0.6% glucose, 0.025 mg/mL insulin, 80 µg/ml apotransferrin, 16 nM progesterone, 60 µM putrescine, 24 nM sodium selenite, and 2% heat-inactivated fetal bovine serum) and maintained for five days.

### Population doubling level (PDL) analysis

PDL analysis was performed in neurosphere-like cultures by seeding NPCs as 5 × 10^4^ cells/cm^2^ in ultra-low attached plates in GM. Cells were disaggregated and quantified every three days, with the process repeated over fifteen passages. The calculation of PDL used the following formula: *n* = log (2)/(log UCY − log *l*), where *n* = the final PDL number at the end of a given subculture, UCY = the cell number counted at that time point, *l* = the cell number seeded to begin that subculture.

### Proliferation assay by BrdU incorporation and Ki67 immune detection

NPCs were dissociated and plated on Matrigel™ coated coverslips at 4 × 10^4^ cells/cm^2^ in GM. 24 h later, the media was replaced with fresh GM containing 10 µM bromodeoxyuridine (BrdU; Roche) for 90 min. Cells were then fixed with 4% PFA for 10 min, washed, and incubated with 2N HCl for 20 min at room temperature. The HCl was then neutralized with 0.1M sodium borate (pH 8.5) for 5 min before the double immunofluorescence assay.

### Immunofluorescence assay

After fixation in 4% PFA, spinal cords were dehydrated and set into paraffin for cross-sectioning into 7 μm slices. NPCs cultures were fixed with 4% PFA for 10 min at room temperature. Tissue slices were de-waxed and re-hydrated before unmasked using Tris–EDTA Buffer (10 mM Tris Base, 1 mM EDTA Solution, 0.05% Tween 20, pH 9.0) at 97 °C for 25 min for antigenic retrieval. Permeabilization and blocking steps were performed at the same time using phosphate buffer saline (PBS) containing 0.5% Triton x-100, 10% fetal bovine serum, and 5% horse serum for tissue slices and PBS containing 0.1% Triton x-100 and 3% normal goat serum for NPCs for 1 h at room temperature. Next, cells or tissues were incubated overnight at 4 °C with the primary antibodies at the indicated dilution: α-mouse III-beta-tubulin (1:400, MO15013, Neuromics), α-rabbit anti-Olig2 (1:400, AB9610, Millipore), α-chicken Ki67 (1:600, ab15580, Abcam), α-mouse anti-BrdU (1:1000, SIGMA), α-rabbit pH2AX (1:800, 2577, Cell Signaling), α-mouse FOXJ1 (1:200, Invitrogen, 4-9965-82), α-chicken glial fibrillary acidic protein (GFAP; 1:1000, PA1-10004, Thermo Fisher), α-rabbit Sox2 (1:300, ab75179, ABCAM), α-chicken NeuN (1:600. ABN91, Millipore), α-rabbit anti-TLR2 (1:50, sc-10738, Santa Cruz Biotechnology), α-mouse TLR4 (1:50, sc-293072, Santa Cruz Biotechnology). After washing in PBS, cells were incubated with Goat anti-mouse secondary antibody conjugated with Alexa-Fluor 488, Goat anti-rabbit secondary antibody conjugated with AlexaFluor-555, or Goat anti-chicken conjugated with AlexaFluor-647 (dilution 1:400 in blocking solution, Invitrogen) for 1 h at room temperature. Finally, nuclei were visualized with 4',6-diamidine-2'-phenylindole dihydrochloride (DAPI; 1:1000, 10 min incubation, Sigma). After additional washes, immune-reactivity was analyzed, and cell images were acquired and analyzed using the Leica IM 500 4.0 image-processing program (Leica, Bensheim, Germany), confocal microscopy (SP2; Leica), or tissue slice scanner Aperio Versa (Leica Biosystems). The quantification of apoptosis using the levels of pyknotic nuclei stained with DAPI used the identification of labeled nuclei exhibiting a size below the average nucleus size and displaying hyper-condensation of chromatin.

GFAP signal intensity was measured using Image J software, and the threshold tool was used to establish the basal signal from WT NPCs. Binarized images with fluorescence intensity above WT threshold, merged with the DAPI signal, were employed to quantify the number of astrocytes with high fluorescence intensity for GFAP in TLR2 and TL4 deficient NPC cultures. Cell quantification analysis used at least fifteen random fields per group in triplicate in three individual cell cultures. For tissue analysis, all data were normalized to the total evaluated area using Image J software.

### RNA isolation and semi-quantitative RT-PCR

Total RNA was isolated from NPCs and homogenized spinal cord tissue using TRIzol reagent (Thermo Fisher Scientific) according to the manufacturer's instructions. 1 μg of total RNA treated with DNaseI (Qiagen) was reverse transcribed using the high-capacity RNA-to-cDNA™ kit (Applied Biosystems) in a total reaction volume of 20 µl through incubation at 37 °C during 120 min using random hexamer primers. Specific target primers were designed using Primer-BLAST (NCBI)—sequences, annealing temperature of 60 °C are detailed in Additional file 1: Table S1. A total of 10 ng of cDNA was used for quantitative PCR in a total volume of 10 µl using AceQ SYBR qPCR Master Mix (1:2, ThermoFisher) on a LightCycler 480 (Roche, Switzerland). Amplification conditions were determined by each pair of primers to present amplification efficiency close to 100% and a single peak in melt-curve analyses. Each real-time PCR reaction was performed in triplicate from at least three independent experiments. Peptidylprolyl Isomerase A (PPIA) was used as a housekeeping gene. The log fold change in mRNA expression was calculated from ΔΔCt values relative to control samples and the ratio to the housekeeping gene expression (2^^ct HK^/2^^ctgene^) [29].

### Western blot analysis

Total protein was extracted from cell cultures using a lysis buffer containing 50 mM Tris–HCl, pH 7.5, 150 mM NaCl, 0.02% NaN3, 0.1 SDS, 1% NP40, 1 mM EDTA, 2 mg/mL leupeptin, 2 mg/ml aprotinin, 1 mM phenylmethylsulfonyl fluoride (PMSF), and 1 × Protease Inhibitor Cocktail (Roche Diagnostics, San Diego, CA, USA). The protein concentrations of the supernatant were determined using (BCA) Protein Assay Kit (Thermofisher Scientific). An equal amount of proteins (30 μg/well) were separated by 10% SDS-PAGE and transferred to a polyvinylidene fluoride (PVDF) membrane. The membrane was blocked with 1% bovine serum albumin in Tris buffered-saline with 0.1% Tween-20 for 1 h at room temperature and incubated at 4 °C overnight with the corresponding primary antibody at the indicated dilution: iNOS (1:600, ab15323, ABCAM), IRF1 (1:300, sc-640, Santa Cruz Biotechnology), pERK (1:1000 4370, Cell Signaling), ERK (1:300, sc-271269, Santa Cruz Biotechnology), TLR2 (1:300, sc-21760, Santa Cruz Biotechnology), TLR4 (1:1000, 19811-1-AP, Proteintech), and STAT3 (1:300, sc-482, Santa Cruz Biotechnology).To ensure loading with equal amounts of protein lysates, blots were probed with antibodies against α-tubulin or β-actin (1:10,000 SIGMA). Signal detection was performed with an enhanced chemiluminescence kit (ECL Plus Western blotting detection reagent—GE Healthcare, Piscataway Township, NJ, USA), and bands developed using Amersham Imager 600. Relative protein expression was quantified using the Image Studio Lite software.

### Statistical analysis

All experimental data were collected from at least three independent in vitro experiments or up to eight different animals for tissue analysis, and results were reported as the mean ± the standard error of the mean (S.E.M.) as indicated for each set of data. For the comparisons between two groups of values, the statistical analysis of the results used the Student's *t*-test for normally distributed data. Results between groups were first assessed for normality using the Shapiro–Wilk test and then analyzed using one-way ANOVA, with appropriate corrections such as Tukey's post hoc tests used for comparisons. Statistical analyses were performed using GraphPad software. Differences were considered significant at *p < 0.05; **p < 0.01; ***p < 0.001 and ****p < 0.0001. All statistics and post hoc tests are stated in the text, and corrections for multiple comparisons performed where appropriate.

## Results

### TLR2 and TLR4 expression maintain the neural progenitor cell population in the postnatal mouse spinal cord

**Fig. 1:**
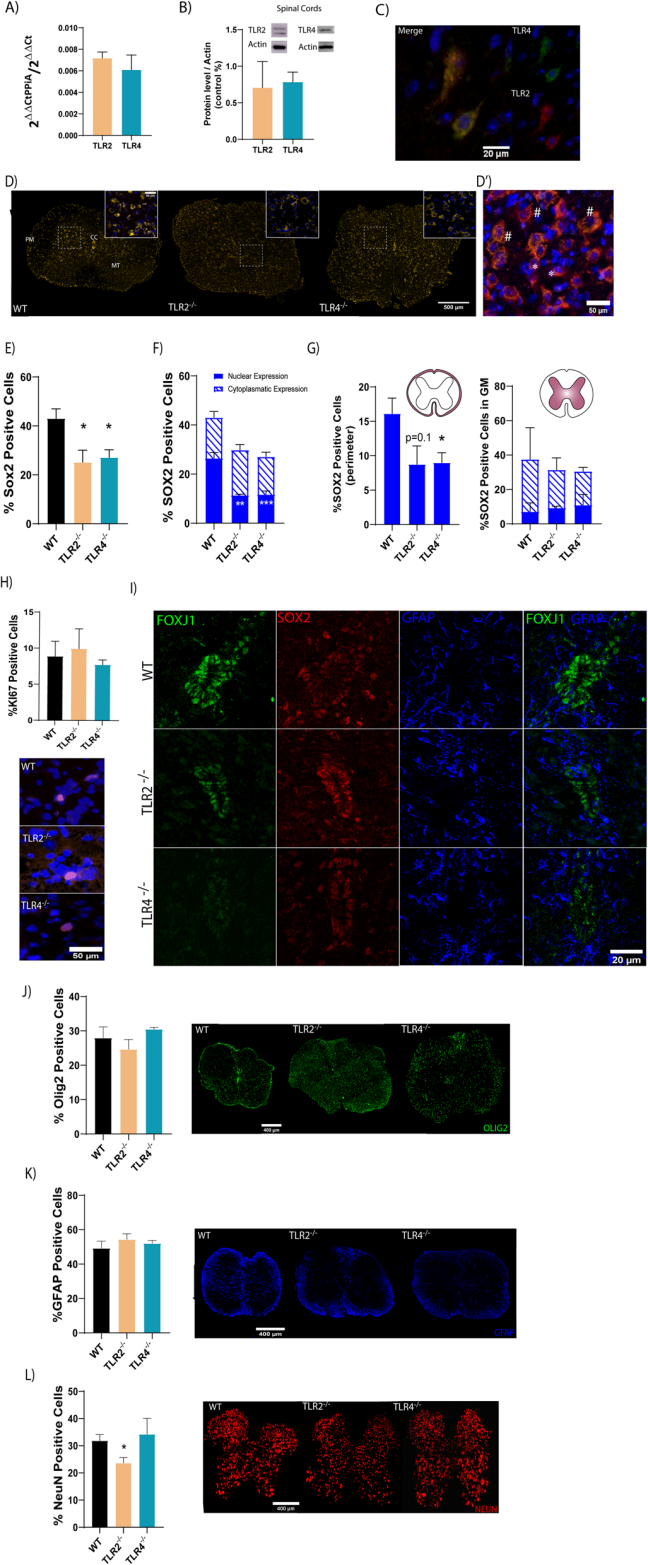
Analysis of spinal cord NPCs from WT and TLR2 and TLR4 knockout mice. **A** Gene expression analysis of TLR2 and TLR4 in the spinal cord tissue of WT mice; **B** representative Western blots and the overall protein levels of TLR2 and TLR4 from WT spinal cord tissue. β-actin was used as a total protein loading control; **C** representative image showing double immunoreactivity (merge) for TLR2 (orange) or TLR4 (green) and DAPI (blue-for nuclei counterstaining) in spinal cord coronal slices. A magnified view of the indicated area with a white square for each staining; **D** representative immunofluorescence images of SOX2 (orange) in spinal cord coronal sections from WT, TLR2^−/−^ and TLR4^−/−^ mice. *Inset:* higher magnification of the indicated area in the square (Sox2, orange; Dapi, blue); D´) Representative image of typical Sox2 nuclear (*) or cytoplasmic (#) sub-cellular expression (orange) found all three samples, with cytoplasmic co-localization with NeuN (green). **E** Quantification of Sox2-positive cells expressed as a percentage of the total number of cells. **F** Quantification of nuclear (full) or cytoplasmic (striped) Sox2-expressing cells as a percentage of the total number of cells in the entire spinal cord area. **G** Quantification of nuclear (full) or cytoplasmic (striped) Sox2-expressing cells as a percentage of the total number of cells in the grey matter (left) and white matter at the PM (right); **H** *upper panel:* quantification of % of cells positive for KI67, *lower panels*: representative images of Ki67 staining; **I** representative immunostaining images of the CC of WT, TLR2^−/−^ or TLR4^−/−^ mouse spinal cords. For each genotype individual and merged staining’s are shown for FOXJ (green), SOX2 (red); GFAP (blue); *Left graphs*: Quantification of Olig2-positive cells (**J**); GFAP (**K**) or NeuN (**L**) expressed as a percentage of the total number of cells stained with DAPI in spinal cord coronal sections; *Right panels:* representative images for each staining. Data shown as mean ± SEM. Results assessed for normality using the Shapiro–Wilk test and one-way ANOVA with Tukey post hoc test; *p < 0.05; **p < 0.01 or ***p < 0.001 vs. WT

TLR2 and TLR4 displayed similar expression levels in mouse neonatal spinal cord extracts at the mRNA (Fig. 1A) and protein levels (Fig. 1B). We also found constitutive mRNA expression of other TLR family members including TLR1, 3, 6, 8 and 9. TLR3 showed the highest expression levels; only this receptor showed to be significantly different in comparison with the other tested TLR members (Additional file 2: Figure S1). The immune-histochemical analysis of spinal cords provided evidence that most TLR2-expressing cells (orange) co-expressed TLR4 (green), as shown in the representative images (Fig. 1C; white square contains higher magnification of the indicated area shown for each staining).

To further investigate the role of TLR2 and TLR4 in neonatal mouse spinal cord-resident NPCs, we explored the expression of Sox2, the earliest transcription factors expressed in neural stem and progenitor cells [30], playing a key role in specifying early neural lineages and brain development [31]. In the early postnatal WT mouse spinal cord, we observed Sox2 expression within the central canal (CC) (Fig. 1D) in cells homogeneously distributed dorsally and ventrally within the grey matter at the mantle area (Fig. 1D; MT) and within the lining along the cord perimeter in the white matter (PM) (Fig. 1D). Interestingly, the deletion of TLR2 or TLR4 significantly diminished the total number of Sox2-positive cells (Fig. 1E). When we closely inspected Sox2-expressing cells, we distinguished two different expression patterns—nuclear Sox2 expression, corresponding to dividing progenitors NPCs [32] (Fig. 1D´, *), previously reported as potential oligodendrocyte progenitor cells (OPC) and mature astrocytes [33], and cytoplasmic Sox2 expression, corresponding to migrating and non-dividing neuroblasts [34] (Fig. 1D´, #). Our analysis revealed that TLR2 or TLR4 loss only affected the number of cells with nuclear Sox2 expression, assigned as the NPCs (Fig. 1F; solid blue bar). We observed the vast majority of cells with cytoplasmic Sox2 expression located in the grey matter i.e., neuroblasts (Fig. 1G, left panel, blue striped bar) co-existing alongside cells with nuclear Sox2 expression (Fig. 1G, left, solid blue bar); however, TLR2 or TLR4 loss failed to impact these Sox2 positive cells. Likewise, all Sox2-positive cells located to the cord perimeter/meningeal zone, [35], displayed nuclear Sox2 expression and here we found a significant impact by the depletion of TLR2 or TLR4 by promoting a significant decrease on the number of this neural precursor population (Fig. 1G, left). Overall, these findings suggest that TLR2 and TLR4 support spinal cord NPC maintenance at early postnatal stages.

An analysis of cell proliferation within the spinal cords of WT and also TLR2^−/−^ and TLR4^−/−^ postnatal mice via Ki67 immunostaining failed to find any significant differences (Fig. 1H), suggesting that the decreased number of Sox2-expressing proliferating cells fails to significantly affect overall proliferative activity at the postnatal stage.

We also explored whether the TLR2 or TLR4 expression contributed to the FoxJ1-expressing ependymal precursor cell population. FoxJ1, a transcription factor involved in ciliogenesis [36], is considered a marker of fully differentiated and ciliated ependymal progenitor cells that line the central canal and divide and differentiate at postnatal time points [37, 38]. However, we failed to find any significant differences in the number of FoxJ1-positive cells in WT, TLR2^−/−^, and TLR4^−/−^ mouse postnatal spinal cords (Fig. 1I, green). FoxJ1 positive cells co-localized with Sox2 (Fig. 1I orange) but not with GFAP (Fig. 1I, blue)[39].

The analysis of total number of early precursors for oligodendrocytes (Fig. 1J) determined by the expression of Olig2, which can generate further olygodendrocytes or motoneurons precursors [40] or astrocytes (Fig. 1K) by the expression of GFAP, did not show significant differences. Finally, an analysis of NeuN levels demonstrated a significant reduction in the total number of neurons in TLR2^−/−^ but not TLR4^−/−^ mouse neonatal spinal cords than WT mice (Fig. 1L).

Overall, these studies prompted us to undertake a more detailed in vitro analysis of in vitro-expanded NPCs isolated from the spinal cords of WT, TLR2^−/−^, and TLR4^−/−^ mice to decipher the specific roles of these two TLRs in NPC self-renewal and differentiation.

### TLR expression by in vitro-expanded NPCs isolated from the postnatal mouse spinal cord

**Fig. 2:**
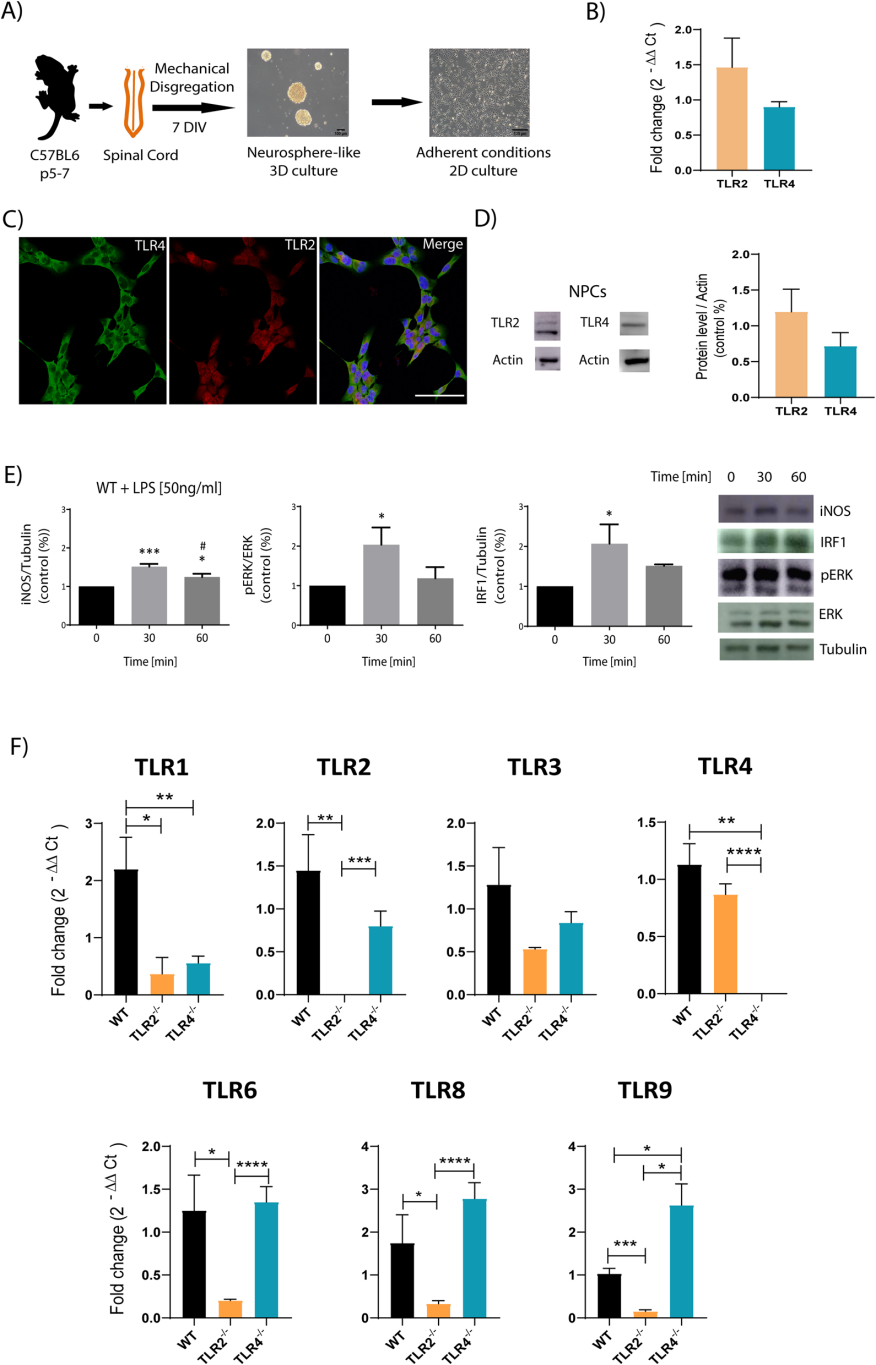
TLR2 and TLR4 expression analysis of in vitro-expanded spinal cord NPCs. **A** Schematic representation of NPC extraction from neonatal mouse spinal cords and their culture in both neurosphere-like 3D and adherent conditions. **B** Gene expression analysis of TLR2 and TLR4 in WT NPCs. **C** Representative confocal images showing the expression of TLR4 (green), TLR2 (red), and the co-expression (merge) in WT NPCs grown under adherent conditions. **D** Representative Western blot and quantification of TLR2 and TLR4 protein expression in WT NPCs grown under adherent conditions. **E** Densitometric analysis Western blot analysis of iNOS, pERK, and IRF1 levels in response to LPS (50 ng/ml) for 0, 30, or 60 min in WT NPCs grown under adherent conditions and a representative Western blot. Data shown as mean ± SEM (n = 4 per group) compared with the control group (0 min) and two-tailed unpaired t-test was used to analyze significant differences; *p > 0.05; ***p < 0.001 vs. 0 min.; **F** gene expression analysis of select TLRs in WT, TLR2^−/−^ and TLR4^−/−^ NPCs. Data shown as mean ± SEM (n = 6 per group) and two-tailed unpaired t-test was used to analyze significant differences; *p > 0.05; **p < 0.01; ***p < 0.001; ****p < 0.0001 as indicated

We next explored the relative expression of TLR2 and TLR4 by in vitro*-*expanded NPCs derived from mouse neonatal spinal cords (schematic representation of the NPC in vitro expansion procedure shown in Fig. 2A). We failed to find significant differences in mRNA (Fig. 2B) and protein (Fig. 2D) expression levels for TLR2 and TLR4 in WT NPCs. Immunofluorescence analysis confirms that all NPCs co-express for receptors (Fig. 2C). Next, we explored MyD88/TRIF-mediated responses of TLRs in WT NPCs upon stimulation with 50 ng/ml of LPS for 30 or 60 min (Fig. 2E). All three tested downstream mediators of TLR2 and TLR4, induced nitric oxide synthase (iNOS), phosphorylated extracellular signal-regulated kinase (pERK), and interferon regulatory factor 1 (IRF1), displayed maximal activation (as measured by an increase in protein levels for iNOS and IRF1 and phosphorylation of ERK) 30 min after stimulation, with a decrease 30 min later (Fig. 2E; representative Western blots shown on the left) as previously described in other cell types [41, 42]. These data provided evidence for the responsive and functional nature of TLR2 and TLR4 to LPS by in vitro*-*expanded WT NPCs derived from mouse neonatal spinal cords.

We also evaluated the expression of the TLR family members in NPCs isolated from the spinal cords of TLR2^−/−^ and TLR4^−/−^ neonatal mice. Our findings confirmed the lack of *TLR2* and *TLR4* expression in TLR2^−/−^ and TLR4^−/−^ mice, respectively, and additionally demonstrated that TLR2 loss significantly reduced the expression level of *TLR1*, *TLR6*, *TLR8*, and *TLR9*, but not *TLR3* and *TLR4*, and that TLR4 deletion significantly reduced the expression of *TLR1* and increased the expression of *TLR9* (Fig. 2F).

### TLR2, but not TLR4, regulates the self-renewal of in vitro-expanded NPCs isolated from the postnatal mouse spinal cord

**Fig. 3:**
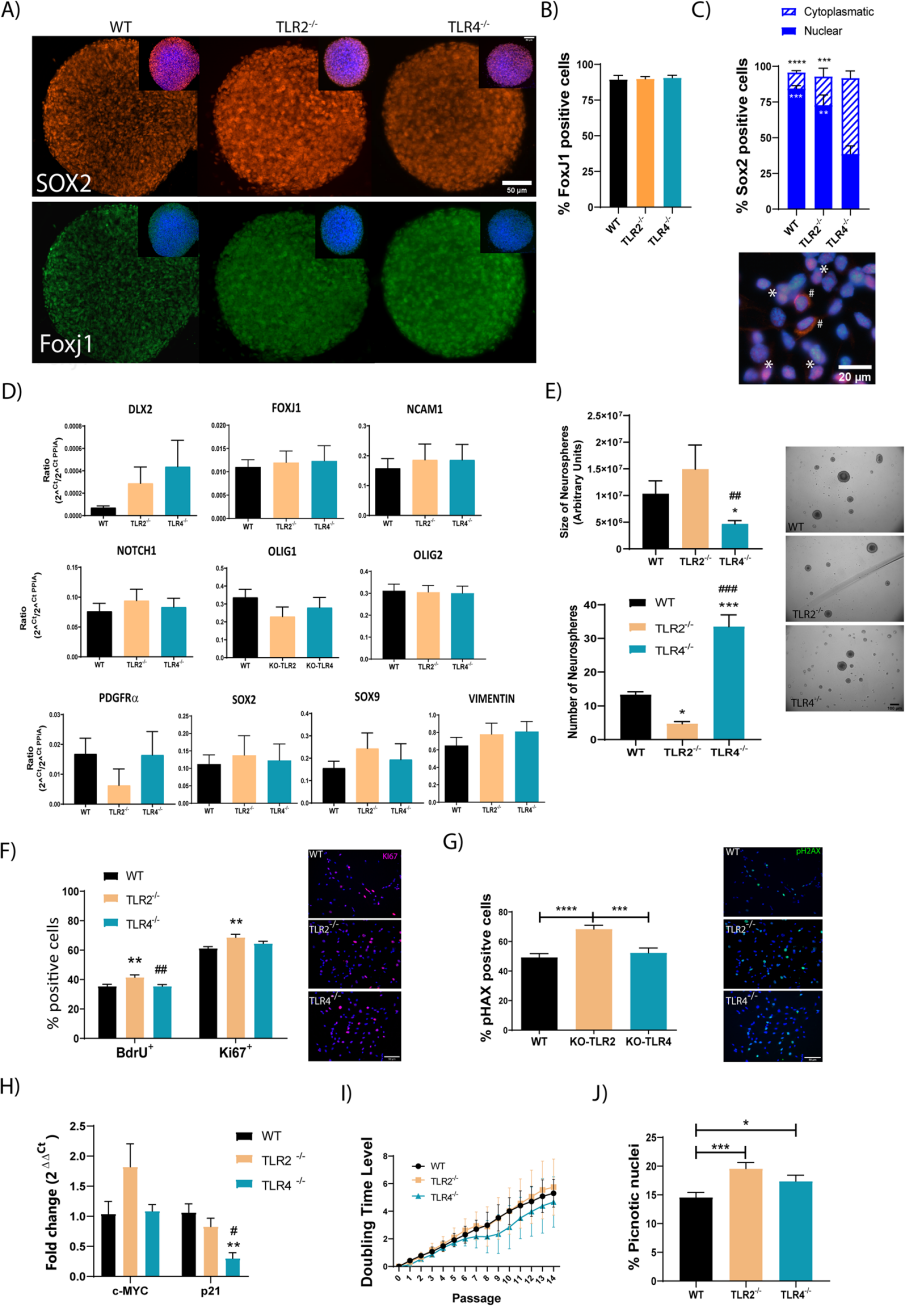
Involvement of TLR2 and TLR4 in the self-renewal of in vitro-expanded spinal cord NPCs. **A** Representative images of immunofluorescence assay for Sox2 (upper panels, orange) and FoxJ1 (lower panels, green) detection in neurospheres formed using WT, TLR2^−/−^ and TLR4^−/−^ NPCs. *Inset*: double staining images for Sox2 (orange) or FoxJ1 (green) with DAPI (blue); **B** Quantification of FoxJ1 positive cells by immunoassay expressed as the percentage of total DAPI positive cells. **C** *Upper graph:* Quantification of Sox2-positive cells represented as nuclear (full) or cytoplasmic expression (striped). Black stars (  ) vs. TLR4^−/−^ for cytoplasmic Sox2 and White stars (  ) vs. TLR4^−/−^ for nuclear Sox2. *Lower panel:* representative image of typical Sox2 nuclear (*) or cytoplasmic (#) expression co-stained with DAPii (blue); **D** Gene expression analysis in WT, TLR2^−/−^, and TLR4^−/−^ NPCs of the indicated genes. **E** Quantification of size (upper graph) and number (lower graph) of neurospheres formed from WT, TLR2^−/−^, and TLR4^−/−^ NPCs 48 h incubation after unicellular disaggregation. * vs. WT; # vs. TLR2^−/−^. Representative images of the indicated neurosphere like cultures are shown (right); **F** (Left) Quantification of BrdU and Ki67 positive cells in WT, TLR2^−/−^ or TLR4^−/−^ NPCs represented as percentages of the total cells. * vs. WT; # vs. TLR2^−/−^. (Right) Representative images of the double immunofluorescence—BrdU (red) and Ki67 (green). **G** (Left) Quantification of H2AX positive cells in WT, TLR2^−/−^ or TLR4^−/−^ NPCs represent as percentages of the total cells. * vs. WT; # vs. TLR2^−/−^. (Right) Representative images of the pH2AX (green) immunofluorescence with DAPI used for nuclei counterstaining (blue) in WT, TLR2^−/−^ or TLR4^−/−^ NPCs. **H** Gene expression by qPCR of cMYC and p21 transcripts in WT, TLR2^−/−^ and TLR4^−/−^ NPCs. **I** Population doubling level analysis in WT, TLR2^−/−^ and TLR4^−/−^ NPCs expressed as the mean of three independent experiments. **J** Quantification of pyknotic nuclei in WT, TLR2^−/−^ and TLR4^−/−^ NPCs from DAPI nuclei staining (identified as smaller than normal with hyper condensate chromatin) represented as a percentage of the total cells. Data shown as mean ± SEM. Results were assessed for normality using the Shapiro–Wilk test and one-way ANOVA with Tukey post hoc test; * or ^#^*p* < 0,05; ** or ^##^ *p* < 0.01; *** or ^###^*p* < 0.001; ****p < 0.0001

We further investigated the role of TLR2 and TLR4 in the self-renewal and proliferation of in vitro-expanded NPCs isolated from the postnatal mouse spinal cord. Isolated and expanded WT, TLR2^−/−^ or TLR4^−/−^ NPCs all expressed Sox2 (Fig. 3A, representative images in orange; 3D, positive cells quantification) and FoxJ1 (Fig. 3A, representative images in green, Fig. 3C, positive cell quantification) to a similar degree (mRNA—Fig. 3B). We again found significant differences regarding Sox2 subcellular location—TLR4^−/−^ NPCs displayed a significantly reduced number of cells with nuclear Sox2 expression compared to TLR2^−/−^ NPCs or WT NPCs (Fig. 3C; graph and representative image showing typical Sox2 nuclear (*) or cytoplasmic (#) expression at each of the samples). This result agrees with the data found in Fig. 1H showing a significant reduction of the Sox2 positive cells at the perimeter SC.

We also evaluated the expression of marker genes preferentially expressed in immature NPCs (Notch1, Sox9, Dlx2, NCAM1, Olig1, PDGFRα, Sox2, FoxJ1 and vimentin) in WT, TLR2^−/−^, or TLR4^−/−^ NPCs to evaluate whether deletion of TLR2 or TLR4 could influence early glial (Sox9, Olig1, PDGFRα) or neuronal determination (Notch1, Dlx2, NCAM1). However, the mRNA expression analysis failed to find any difference in expression for the noted genes suggesting that TLR2 and TLR4 have not significantly influence on these early NPC multilineage markers at this early postnatal stage (Fig. 3D).

We next evaluated the ability of TLR2^−/−^ and TLR4^−/−^ NPCs to form primary neurospheres to explore their self-renewal capacity [43]. Overall, TLR2^−/−^ NPCs formed significantly larger but less numerous neurospheres when compared to WT and TLR4^−/−^ NPC-derived neurospheres (Fig. 3E), indicating the preferential formation of primary neurospheres and enhanced self-renewal. Meanwhile, TLR4^−/−^ NPCs formed significantly smaller and more numerous neurospheres (Fig. 3E), indicating the more rapid formation of secondary neurospheres and limited self-renewal.

We also studied proliferation via BrdU incorporation and Ki67 immunostaining in WT, TLR2^−/−^, and TLR4^−/−^ NPCs grown under adherent conditions, finding that only TLR2 deletion significantly increased NPC proliferation (Fig. 3F). Analysis of phospho-H2AX levels, which mark cells undergoing mitotic stress [44], revealed a significantly higher number of positive cells in TLR2^−/−^ NPCs when compared to WT and TLR4^−/−^ NPCs (Fig. 3G, representative images, right and graph, left). TLR2^−/−^ NPCs also displayed a higher level of cMyc gene expression than TLR2^−/−^ NPCs (Fig. 3H, left), indicative of enhanced cell cycle activity [45]. Analysis of p21 expression found significantly lower gene expression levels in TLR4^−/−^ NPCs than WT NPCs, suggestive of a potentially deregulated cell cycle (Fig. 3H, right); however, PDL analysis suggested a slow-down in growth for TLR4^−/−^ NPCs only (Fig. 3I).

Interestingly, both TLR2^−/−^ and TLR4^−/−^ NPCs exhibited increased apoptosis than WT NPCs. The significant increase in cell death could balance the increased proliferation in TLR2^−/−^ NPCs to explain the lack of PDL differences compared to WT NPCs. As TLR4^−/−^ NPCs did not display higher proliferative rates, the significantly increased apoptosis rate may explain the observed reduction in PDL (Fig. 3J).

Taken together, our data suggest that TLR4 maintains in vitro-expanded NPCs isolated from the postnatal mouse spinal cord in a proliferative and undifferentiated state., while TLR2 expression limits NPC proliferation and self-renewal.

### TLR2 and TLR4 in differentially contribution to the formation of mature neurons and glial cells

**Fig. 4:**
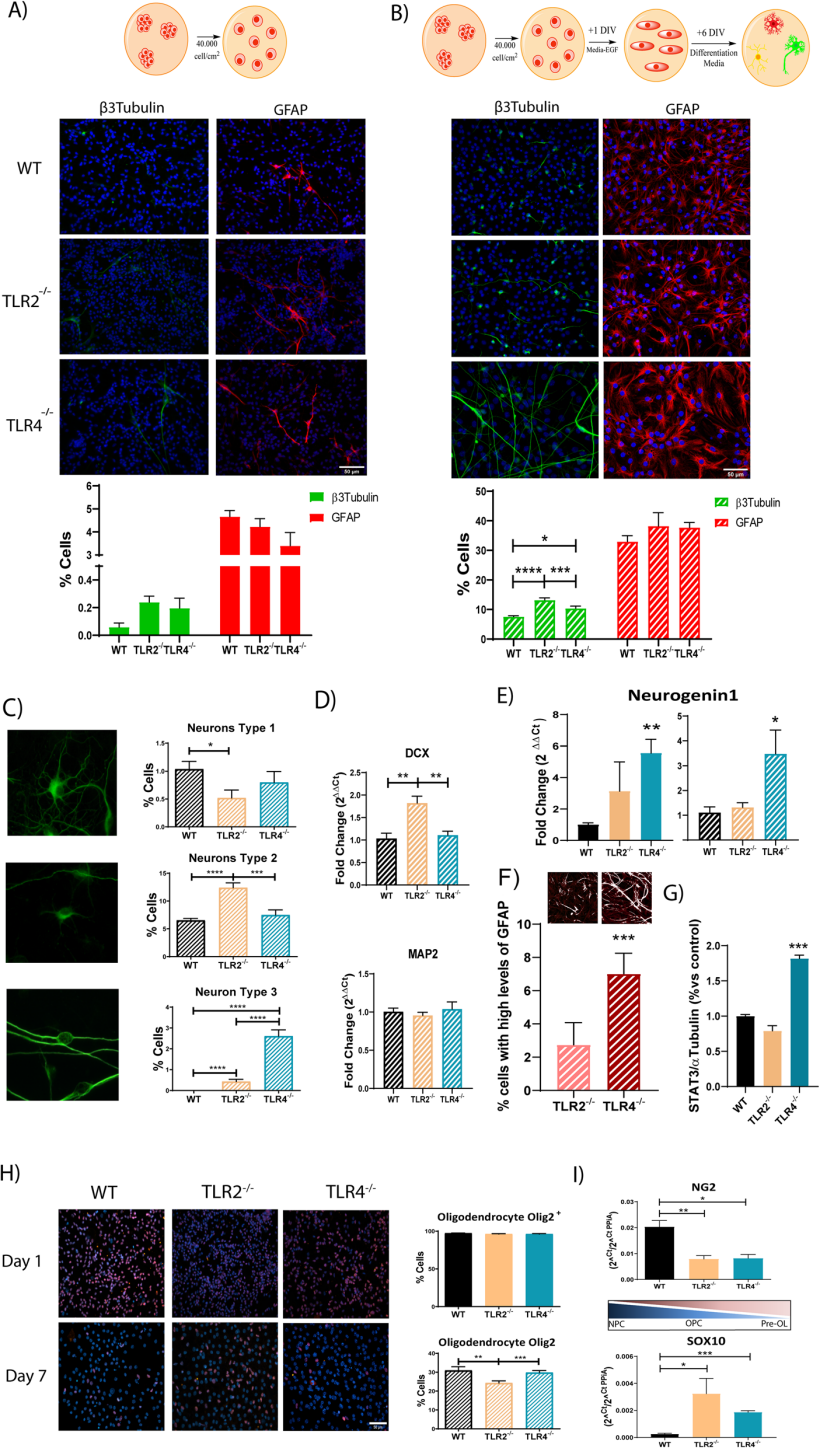
TLR2 and TLR4 loss influence the spontaneous differentiation of in vitro-expanded spinal cord NPCs. **A**, **B** Representative immunofluorescence images of β3tubulin (neural marker; green) and GFAP (astrocytic marker, red) and the corresponding quantification data of the percentage of positive cells (lower panels) in NPCs grown in growth medium (**A**) or in differentiation medium (**B**) as summarized in the diagrams (top images). DAPI used for nuclei counterstaining (blue). **C** Morphological classification of the three distinct types of neurons identified from β3tubulin staining—type 1 (pyramidal-like cells; upper panel), Type 2 (rounded, with no cell expansions; central panel), and Type 3 (bipolar cells; lower panel). Quantification and comparative analysis of the percentage of the corresponding type of neurons shown for WT, TLR2^−/−^, and TLR4^−/−^ NPCs; **D** Gene expression analysis of Dcx (early neuronal marker, upper graph) and MAP2 (late neuronal marker, lower graph). **E** Gene expression analysis of Neurogenin1 in growth medium (left) or differentiation medium (right) in WT, TLR2^−/−^ and TLR4^−/−^ NPCs. **F** (lower graph) Quantification and comparative analysis versus WT NPCs of the number of cells expressing higher GFAP protein expression levels in TLR2^−/−^ and TLR4^−/−^ cells; (upper panels) Binarized representative images for TLR2^−/−^ and TLR4^−/−^ NPCs showing cells with a fluorescence intensity above the WT levels threshold. **G** Protein expression levels of STAT3 in WT, TLR2^−/−^ and TLR4^−/−^ NPCs (α-tubulin as a loading control). **H** (Left) Representative images of immunofluorescence staining of Olig2 (orange) in NPC in growth medium (upper panels, day 1) and differentiation medium (lower panels, day 7). DAPI used for nuclei counterstaining. (Right) Quantitative analysis of Olig2 positive cells at one day (upper graph) and seven days (lower graph). **I** Gene expression analysis for NG2 (upper graph) and SOX10 (lower graph). Data shown as mean ± SEM. Results were assessed for normality using the Shapiro–Wilk test and one-way ANOVA with Tukey post hoc test. *p < 0.05; **p < 0.01; ***p < 0.001

We finally sought to evaluate the contribution of TLR2 or TLR4 to glial or neuronal cell-fate determination by seeding WT, TLR2^−/−^ or TLR4^−/−^ NPCs onto Matrigel™ coated plates and analyzing comparing their spontaneous differentiation one day (undifferentiated) or seven days (spontaneously differentiated) after growth factor withdrawal, as indicated by the experimental schemes (Fig. 4A, B, top). We failed to encounter any differences in neuronal (β3Tubulin) (Fig. 4A, left panels, green) or astrocytic (GFAP) (Fig. 4A, right panels, red) differentiation of NPCs as a consequence of TLR2 or TLR4 loss after one day (undifferentiated cell condition); however, we encountered significant differences in the number of neurons (Fig. 4B, left panels) but not astrocytes (Fig. 4B, right panels) after induced differentiation as a consequence of TLR2 or TLR4 loss after seven days. TLR2^−/−^ NPCs displayed a highly significant increase in neuronal differentiation compared to WT and TLR4^−/−^ NPCs, while TLR4^−/−^ NPCs displayed significantly higher neuronal differentiation than WT NPCs (Fig. 4B, left panels).

Interestingly, the β3Tubulin-expressing neuron-like cells exhibited three different morphologies in all three genotypes (Fig. 4C). Type 1 cells are pyramidal-like neurons, with a prominent soma and numerous dendrites (a morphology compatible with mature neuron); Type 2 are immature-like neurons, which are small, with few neurites (a morphology compatible with undifferentiated neurons); and Type 3 cells were bipolar with very long axonal projections (a morphology compatible with mature neurons). TLR2^−/−^ NPCs preferentially differentiated into Type 2 cells (immature neurons) and expressed elevated levels of *Dcx*, a marker of very early NPCs (Fig. 4D, upper graph). Meanwhile, TLR4^−/−^ NPCs preferentially differentiated into Type 3 cells (mature neurons) but did not express higher levels of *Map2*, a marker of mature neurons (Fig. 4D, lower panel), perhaps indicating a transitional stage of maturation.

We next studied the neural cell fate identity of WT, TLR2^−/−^ or TLR4^−/−^ NPCs by analyzing *Neurogenin1* expression levels [46]. Both self-renewing and spontaneously differentiated NPCs from TLR4^−/−^ NPCs expressed higher levels of *Neurogenin1*) than WT and TLR2^−/−^ NPCs (Fig. 4E), indicating a primed stage of NPCs for neuronal maturation. Overall, TLR2 loss prevented neuronal maturation, while TLR4 loss enhanced neuronal maturation.

We failed to find any differences in the percentage of astrocytic cells, based on the positive reactivity of GFAP, following analysis of WT, TLR2^−/−^, or TLR4^−/−^ NPCs at one day or seven days after induced spontaneous differentiation (Fig. 4A, B); however, we discovered a significant increase in the number of cells with higher GFAP protein expression in TLR4^−/−^ NPCs cultures (Fig. 4F) previously associated to a reactive astrocytic phenotype [47]. Analysis of the Stat3 protein, previously described to be involved in astrocytic reactivity [48, 49], revealed a significant increase in TLR4^−/−^ NPCs compared with WT or TLR2^−/−^ NPCs (Fig. 4G), which could explain the reactive phenotypic profile found in the absence of TLR4.

Analysis of oligodendrocyte differentiation in suitable growth-supporting medium found that WT, TLR2^−/−^ and TLR4^−/−^ NPCs all expressed similar levels of Olig2, a transcription factor expressed in early to mature stage oligodendrocytes [50] (Fig. 4H, upper panels); however, after seven days of spontaneous differentiation, the absence of TLR2 significantly reduced the generation of Olig2 positive cells from NPCs (Fig. 4H, lower panels). We also found the significantly higher expression of Sox10, a transcription factor expressed in early OPCs [51], and the significantly lower expression of NG2, a factor expressed by mature OPCs [52], in the absence of TLR2 or TLR4. Overall, this data also suggests a critical role for TLR2 and TLR4 in oligodendrocyte maturation.

## Discussion

In addition to their ability to respond to the innate immune system, TLRs, including TLR2 and TLR4 which has been more extensively studied, play fundamental roles in cell fate and cell maturation of NPCs in the developing and adult mammalian brain [3]. In our exploration of TLR expression in the neonatal mouse spinal cord, we discovered that TLR2 and TLR4 help to maintain the NPC population and influence cell fate determination in a cell-autonomous manner. Based on the spontaneous capacity of the NPCs lacking of TLR2 to restrict neuronal maturation or the NPCs lacking TLR4, which induce it, and also based on the expression of *Neurogenin1* overexpressed in the lack of TLR4, we propose that expression of TLR2 could promotes and TLR4 could restricts neural maturation, respectively.

During early postnatal stages, at a point when the mouse spinal cord remain incompletely differentiated, Sox2-expressing NPCs exist throughout the spinal cord (in the grey and white matter) lining the meninges and in the ependymal canal, where they co-express FoxJ1, representing the neurogenic niches [38]. NPCs continuously express Sox2 until adulthood to maintain self-renewal capacity by regulating the expression of crucial genes; however, a shift of Sox2 location from the nucleus to the cytoplasm promotes their differentiation [53, 54]. Hyper-acetylation of Sox2 prompts translocation into the cytoplasm and then the differentiation of NPCs [55]. We found that NPCs with nuclear Sox2 expression primarily located to the grey matter of the spinal cord and co-expressed the neuronal marker NeuN but lacked Ki67 expression (data not shown), indicating the existence of a transitional differentiation stage. Despite a significant reduction in the total number of Sox2-positive cells following the loss of TLR2 or TLR4, underlying a role of both receptors in this transitional differentiation stage. The proportion of NPCs with cytoplasmic Sox2 remained unchanged, indicating a relevant and restrictive influence of both receptors at the undifferentiated stages when Sox2 retains its transcriptional activity. Since Sox2 positive precursors are actively dividing, we also expected a reduction in the acutely number of mitotic cells, positive for ki67, however, neither TLR2 or TLR4 deletion significantly influenced over a global proliferating rate in vivo at the neonatal stage, nevertheless, this effect deserves further investigation.

To further evaluate the novel functions of TLR2 and TLR4 within the neurogenic niche of the neonatal spinal cord, we performed further analysis in the isolated and in vitro-expanded NPCs. NPCs from postnatal or adult stages that form neurosphere 3D-like cultures retain their self-renewal and multipotent capacity [43], even taking the form of a pinwheel-like cytoarchitectural structure that mimics the in vivo organization of the endogenous neurogenic niche [57]. NPCs within neurosphere-like cultures maintain the expression of crucial markers such as Sox2 and FoxJ1 and self-renewal/cell differentiation capacities. We found that all NPC expanded in the presence of mitotic enriched media expressed both receptors, TLR2 and TLR4, although not all Sox2 positive cells identified in vivo showed to be immune-reactive for both receptors (data non-shown). We hypothesized that the induced expression of both TLRs in the NPC cultures would be due to the mitotic enriched media; however, this issue needs to be further explored to decipher the regulation of the TLR receptors expression in the precursors cells in culturing conditions. Our analysis in neurosphere-like cultures found evidence that TLR4- but not TLR2^−/−^ NPCs reproduced in vivo findings*,* showing a significant decrease in the percentage of nuclear Sox2-expressing cells. Additionally, a reduction in the proliferation of TLR4^−/−^ NPCs suggested a control on the self-renewal profile of the progenitors, validating the critical role of TLR4 in maintaining mouse neonatal spinal cord NPCs in an undifferentiated state. However, TLR2^−/−^ NPCs, which formed bigger primary neurospheres and possessed higher mitotic activity, also displayed significantly higher rates of apoptosis, suggesting enhanced self-renewal of the progenitor’s population in the absence of this receptor. The increased level of apoptosis could also explain the reduced percentage of Sox2-positive spinal cord NPCs in the absence of TLR2, which would not be compensated by the significant, although modest, increase in mitosis. In a related study, TLR2 deletion in the adult hippocampus under physiological conditions affected cell-fate decisions but not self-renewal, whereas TLR4 loss affected both proliferation and differentiation of NPCs [7]. In retinal precursors as shown in a previous study, indicated that TLR4 deletion increases the proliferation [56]. All of these previous evidences suggest the existence of specific mechanisms for the TLR2 and TLR4-mediated regulation of NPCs in the postnatal mouse spinal cord.

Downstream TLR signaling, activated by distinct ligands for each different member, displays different consequences on NPC maturation. For instance, immortalized human NPCs expressing TLR4 respond to LPS-mediated antagonism by significantly decreasing proliferation and survival, indicating that TLR4 contributes to self-renewing upon exogenous and endogenous activation [20]. The same study linked TLR4 expression and activation to neuronal and oligodendroglial differentiation [20], in agreement with our findings in mouse NPCs. Also, in agreement with previous reports [20, 58], we linked TLR2 and TLR4 expression to OPC maturation in physiological conditions. NPCs lacking TLR2 or TLR4 retain an undifferentiated stage by expressing elevated levels of Sox10, which is associated with early oligodendrocyte commitment, and lower levels of NG2, which is expressed at later stages during OPC maturation, indicating a redundant role of both receptors on oligodendroglia inducing differentiation.

Deletion of TLR2 resulted in a significant reduction of TLR1, 6, 8 and 9, all of them mediates their downstream signaling through MyD88 activation, therefore, the phenotypes found in TLR2 deleted NPC most probably would be reproduced by MyD88 deletion. Indeed, Rolls A and colbs. described that the activation of both TLRs on adult hippocampal NPCs was mediated via MyD88 and induced PKCalpha/beta-dependent activation of the NF-kappaB signaling pathway [7]. TLR4 deletion also significantly downregulated TLR1 expression. Since mouse neonatal spinal cord derived NPC showed high expression level of TLR1 and since this receptor has been shown to participate either in cell differentiation [59] and in cell proliferation [60], we hypothesized that TLR1 would assumed the phenotypes described in TLR2 and TLR4 deleted NPCs. However, this fact needs to be further experimentally explored. We know little regarding the precise cellular and molecular mechanisms involving TLRs in the interplay between immune cells and NPCs. Complex cell-autonomous and non-cell-autonomous mechanisms operate concomitantly with the involvement of TLR-dependent signaling [61, 62]. While studies have demonstrated that immune-deficient mice, with limited TLR activation, show impaired neurogenesis and that specific T helper cells, TLR activators, promote neurogenesis in the adult hippocampus [63], TLR4 has been linked to spontaneous oligodendrocyte maturation and remyelination after damage in an inflammatory environment, such as after SCI [27]. Well-orchestrated adaptive immune responses can also support/induce oligodendrogenesis and neurogenesis. Microglia activated in response to Interleukin-4 support oligodendrogenesis, whereas interferon (IFN)-γ-activated microglia showed a bias towards neurogenesis [64]. Of note, neuron-enriched cultures promote oligodendrogenesis rather than astrogliogenesis, indicating that neurons, and not exclusively immune cells within the neurogenic niche, create the local microenvironment [65]. Here we show that TLR4 deletion in NPCs promotes neurogenesis during spontaneous differentiation; however, whether cell–cell interaction or the release of specific factors contributes to cell fate maturation remains unknown.

The Jak/Stat axis plays a pivotal role in NPC astrocytic differentiation, reactivity [66], and survival [49] and neuronal differentiation [67]. At early developmental stages, Jak/Stat pathway components drives the neurogenic phase, when astrocytic genes are still silent [67]. Proneural proteins such as Neurog1, a bHLH protein, play an essential role in this process, as their deletion causes precocious astrocyte differentiation and limited neural cell fate [68]. We found that the absence of TLR4 prompts an increase in both Neurog1 and Stat3 expression, which prompted neural differentiation and astrocytic activation. The STAT3-mediated neurogenic-to-astrogenic fate switch has been previously described in cultured NPCs [69] (reinforcing the idea of cell-intrinsic programs [70]).

## Conclusions

We found a ligand and microenvironment-independent program that regulates neural precursor cells population maintenance and the neural differentiation at the neonatal stages involving TLR2 and TLR4 depending-signaling and the constitutive expression of Neurogenin1. Thus, TLR signaling regulation could represent a promising avenue to increase cellular plasticity and promote neural differentiation in the spinal cord.

## Supplementary Information

**Additional file 1. Table S1:** Primers sequence used for quantitative RT-PCR.

**Additional file 2. Figure S1:** TLR mRNA expression in the neonatal spinal cord tissue.

## Data Availability

The datasets used and/or analyzed during the current study are available from the corresponding author on reasonable request.

## Abbreviations

BCA: Bicinchoninic acid
BDNF: Brain-derived neurotrophic factor
bFGF: Basic fibroblast growth factor
BrdU: Bromodeoxyuridine
DAMP: Damage-associated patterns
DAPI: 4′,6-Diamidine-2′-phenylindole dihydrochloride
DM: Differentiation medium
EDTA: Ethylene diamine tetra-acetic acid
EGF: Epidermal growth factor
ERK: Extracellular signal-regulated kinases
GFAP: Glial fibrillary acidic protein
GM: Growth medium
IFN: Interferon
iNOS: Inducible nitric oxide synthase
IRF: Interferon regulatory factor
LPS: Lipopolysaccharide
MAP: Microtubule-associated protein
MYD88: Myeloid differentiation primary response 88
NCAM: Neural cell adhesion molecule
NGF: Nerve growth factor
NPC: Neural progenitor cell
OPC: Oligodendrocyte precursor cell
PAMP: Pathogen-associated patterns
PBS: Phosphate buffer saline
PDGFR: Platelet-derived growth factor receptor
PDL: Population doubling level
PFA: Paraformaldehyde
PMSF: Phenylmethylsulfonyl fluoride
PPIA: Peptidylprolyl Isomerase A
RT-PCR: Real-time polymerase chain reaction
SCI: Spinal cord injury
SEM: Standard error of the mean
SOX: SRY-box transcription factor
STAT: Signal transducer and activator of transcription
TLR: Toll-like receptor
TRIF: TIR-domain-containing adapter-inducing interferon
WM: Washing medium
WT: Wild type
