# Supplementary material for: Toll-like receptors 2 and 4 differentially regulate the self-renewal and differentiation of spinal cord neural precursor cells
Source: Stem Cell Res Ther. 2022 Mar 21;13:117. doi: 10.1186/s13287-022-02798-z (PMC8935849; doi:10.1186/s13287-022-02798-z)
Supplement: Supplementary file 1 — Additional file 1. Table S1: Primers sequence used for quantitative RT-PCR. [file 13287_2022_2798_MOESM1_ESM.pdf]

Supplementary Table 1

GENE	FW 5'-3'	RV 5'-3'
3βTUBULIN	TGAAGTCAGCATGAGGGAGA	GCCTGAATAGGTGTCCAAA
OLIG2	ATGGAGAGATGCGTTCGTTC	CCAGACAGGGAGTCAATCTTT
OLIG1	CCCACCTGTTTAGAGCCAGA	AAGCATGCCAGGAAACCAAG
GFAP	AAGGTTGAATCGCTGGAGG	AGTCGTTAGCTTCGTGCTTG
NOTCH1	GAAAGAGGGCATCAGAGGGT	CTTCGCACCTCCCTCCATT
NCAM1	CCGGTTCATAGTCCTGTCCA	CATTCACGATGCTCTGTCTGG
SOX2	TTACCTCTTCCTCCCACTCCA	CCCTCCCAATTCCCTTGTAT
SOX9	CAAGAACAAGCCACACGTCA	GTGGTCTTTCTTGTGCTGCA
SOX10	TGGGCAAGGTCAAGAAGGAA	TTGTGGAGGTGAGGGTACTG
C-MYC	TGAAGAACCAGAGAAGCCCA	CCTATTCAGCACGCTTCTCC
NG2	ACAGCTCCTGCCTCCTTCTTC	TCAACAGACAGCACAGCCCAG
TLR1	ATGATTCTGCCTGGGTGAAG	TCTGGATGAAGTGGGGAGAC
TLR2	CTCCCACTTCAGGCTCTTTG	AGGAACTGGGTGGAGAACCT
TLR3	AGCTTTGCTGGGAACTTTCA	GAAAGATCGAGCTGGGTGAG
TLR4	AAGAGCCGGAAGGTTATTGTG	CCCATTCCAGGTAGGTGTTTC
TLR6	ACACAATCGGTTGCAAAACA	GGAAAGTCAGCTTCGTCAGG
TLR8	GGCACAACTCCCTTGTGATT	CATTTGGGTGCTGTTGTTTG
TLR9	GCTTTGGCCTTTCACTCTTG	AACTGCGCTCTGTGCCTTAT
PPIA	CGCGTCTCCTTCGAGCTGTTT	TGTAAAGTCACCACCCTGGCACA
DLX2	TGGGCTCCTACCAGTACCAC	TGGCTTCCCGTTCACTATTC
PDGFRα	ACGTTCAAGACCAGCGCGAGTT	GCAGCACATTCATACTCTCCA
NEUROG1	GACAGACGGACAGGAGGTTT	ACATCACTCAGGAGACCAGC
P21	GCCTTAGCCCTCACTCTGTG	AGGGCCCTACCGTCCTACT
CYCLIN D1	AGTGCGTGCAGAAGGAGATT	CACAACTTCTCGGCAGTCAA
FOXJ1	TTGACTGGGAGGCCATCTTT	AGGAAGGATGTGGCCAAGAA
