# Supplementary figures and images for: Toll-like receptors 2 and 4 differentially regulate the self-renewal and differentiation of spinal cord neural precursor cells
Source: Stem Cell Res Ther. 2022 Mar 21;13:117. doi: 10.1186/s13287-022-02798-z (PMC8935849; doi:10.1186/s13287-022-02798-z)

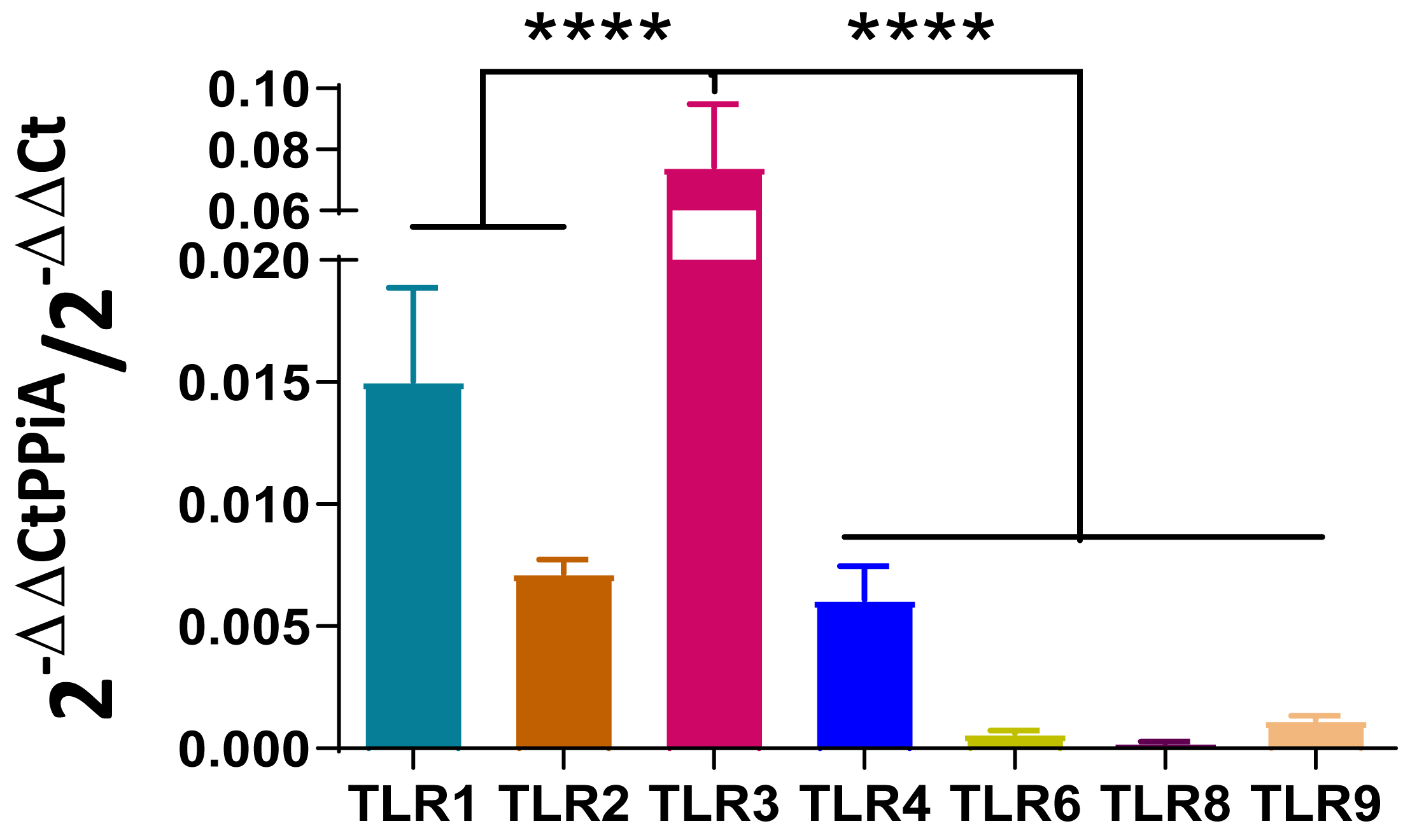

Supplement: Supplementary file 2 — Additional file 2. Figure S1: TLR mRNA expression in the neonatal spinal cord tissue. [file 13287_2022_2798_MOESM2_ESM.tif]
